# Impact of early-onset fetal growth restriction on the neurodevelopmental outcome of very preterm infants at 24 months: a retrospective cohort study

**DOI:** 10.1186/s12887-023-04361-y

**Published:** 2023-10-26

**Authors:** Mariana Cortez Ferreira, Joana Mafra, Ana Dias, Isabel Santos Silva, Adelaide Taborda

**Affiliations:** 1 Neonatology Department, Maternidade Bissaya Barreto, Centro Hospitalar e Universitário de Coimbra, Coimbra, Portugal; 2 Obstetrics Department, Maternidade Bissaya Barreto, Centro Hospitalar e Universitário de Coimbra, Coimbra, Portugal

**Keywords:** Fetal growth restriction, Neurodevelopmental disorder, Preterm infants, Neonates, Children

## Abstract

**Background:**

The association between fetal growth restriction (FGR) and childhood neurodevelopmental delay is unclear and the evidence available to the present date shows conflicting results. Our aim was to analyse the impact of early-onset FGR on the neurodevelopmental outcome at 24 months of corrected age in very preterm infants.

**Methods:**

Retrospective cohort study of very preterm infants (≤ 32 weeks’ gestation) admitted to a neonatal intensive care unit between 1 January 2013–31 December 2019. The control group comprised appropriate for gestational age (AGA) newborns. Griffiths III Mental Development Scale was performed at 24 months of corrected age.

**Results:**

132 infants were included: 44 FGR and 88 AGA. Mean Global Development Quotient (GDQ) was lower for FGR infants (p = 0.004) even after adjusting for maternal and perinatal factors (β_adjusted_ -16.703; p = 0.009). The average scores for the neurodevelopmental domains were highest for personal-social-emotional skills (107.02 ± 16.34), followed by eye/hand coordination (105.61 ± 14.20) and foundation of learning skills (102.23 ± 13.74) and were lowest for gross motor (97.90 ± 11.88) and language/communication skills (96.39 ± 18.88). FGR had a significant negative impact on all domains except for gross motor skills. After adjustment, FGR continued to have a significant adverse impact on language/communication (β_adjusted_ -21.924; p = 0.013), eye/hand coordination (β_adjusted_ -15.446; p = 0.015) and foundation of learning skills (β_adjusted_ -15.211; p = 0.013).

**Conclusions:**

In very preterm infants, FGR was associated with a significantly increased risk of poor neurodevelopmental outcome at 24 months of corrected age compared to age-matched AGA infants.

## Introduction

Advances in neonatal intensive care have improved the survival of severe preterm infants, including those near the limit of viability. [1–4] Nevertheless, the impact on neonatal and long-term morbidity continues to be limited and neurodevelopmental impairment among the survivors appears to have remained stable. [3, 4].

Fetal growth restriction (FGR) is the failure of a fetus to achieve its biological growth potential due to impaired placental function. [5] The definition of FGR varies between different guidelines. The criteria proposed by an international Delphi consensus in 2016 is currently the most accepted definition. [5–7].

FGR occurs in 5–7% of all pregnancies [8, 9] and is estimated to be present in 15–20% of the infants admitted to Neonatal Intensive Care Units (NICU). [10] FGR is a proven risk factor for poor prognosis in very preterm infants, with higher morbidity and mortality rates during the perinatal and neonatal periods. [5–10].

The association between FGR and childhood neurodevelopmental delay is less clear. [10–12] Previous studies have yielded conflicting results. While some papers have reported worse neurodevelopment in FGR infants, [8, 11, 13–15] other authors have reported no link between the two. [9, 10, 12, 16, 17] This discrepancy may be justified by the significant heterogeneity in the studies evaluating neurodevelopment in this population, [16, 18] that ranges from inconsistencies in the definition of FGR and its frequent interchangeability with small for gestational age (SGA) infants, to different methods used to evaluate neurodevelopment. [8, 9] Moreover, some studies do not take into account several perinatal and neonatal characteristics that could also influence neurodevelopment and should be considered as possible confounding factors. [10, 13, 19].

This study aims to analyse the impact of well-defined early-onset FGR on the neurodevelopmental outcome at 24 months of corrected age in very preterm infants and to investigate if its effect is similar across the different neurodevelopmental areas.

## Materials and methods

### Study design and patient selection

We conducted a retrospective cohort study of all preterm infants born with gestational age equal to or under 32 weeks admitted consecutively to the NICU in a tertiary maternity hospital from January 2013 to December 2019. Sample size was calculated using the Fleiss formula with continuity correction and taking into account the results of previous studies. [20, 21] Using an alpha of 0.05 and 80% power, we calculated a minimum sample size of 65 (minimum of 22 with FGR and 43 without FGR).

According to the institution’s protocol, all very preterm infants were included in a follow-up program with frequent reassessment after discharge. This follow-up program was conducted by a specialized multidisciplinary team that includes pediatricians, nurses, and trained educators. At 24 months of corrected age, all children were evaluated for neurodevelopmental outcomes using the Griffiths III Mental Development Scale (GMDS-III). [22] This scale was applied to all very preterm infants by the same two educators who were blind to the diagnosis of FGR and is validated for the Portuguese population. [23].

The control group comprised two appropriate for gestational age (AGA) newborns for every FGR infant. These neonates were matched for gestational age and were admitted to the NICU immediately before or after the FGR infant. Twin infants with FGR were paired with neonates with the same chorionicity and amnionicity. In the event only one twin had FGR, the other one was automatically included in the control group.

Neonates with major congenital malformations or genetic diagnoses that cause lifelong impact were excluded. Children who were lost to follow-up or died before 24 months of corrected age were also excluded.

Clinical data was obtained through the review of the perinatal and neonatal medical records included in the NICU database, the prospective National Registry of Very Preterm Newborns and the eNewborn European Network database, and through the follow-up assessment registered in the personal clinical file.

### Data collection

FGR was diagnosed using the international Delphi consensus: onset before 32 weeks of gestation of an absent end-diastolic flow in the umbilical artery or a fetal abdominal circumference or estimated fetal weight below the 3rd centile or below the 10th centile combined with abnormal Doppler findings in uterine or umbilical arteries. [5].

Sociodemographic characteristics (age, parity, education level) and maternal morbidity factors (placenta previa, chorioamnionitis, hypertension/preeclampsia, HELLP (hemolysis, elevated liver enzymes, and low platelets) syndrome, gestational diabetes, TORCH (toxoplasmosis, rubella, cytomegalovirus, and herpes simplex virus) screening, thrombophilia, autoimmune disease and tobacco use) were included in the analysis. Gestational age was estimated using the first trimester ultrasound.

Perinatal factors such as pregnancy surveillance, multifetal gestation, antenatal corticosteroid therapy for pulmonary fetal maturation, antenatal magnesium sulphate administration for fetal neuroprotection, labor induction, prolonged premature rupture of membranes (PPRM), cesarean delivery, outborn status, sex, gestational age, birthweight, five-minute Apgar score less than 7 and endotracheal intubation during neonatal resuscitation were also retrieved.

Neonatal characteristics and morbidity were also explored. Clinical risk index for babies (CRIB) was assessed. [24] Late-onset sepsis (LOS) was defined as clinical sepsis and abnormal laboratory findings (leukocyte count above 30,000/µL or under 5000/µL and C-reactive protein above 2 mg/dL), irrespective of blood culture results. [25] Bronchopulmonary dysplasia (BPD) was defined as oxygen need at 36 weeks postmenstrual age. [26] Patent arterial duct was systematically evaluated by echocardiogram according to protocol or in case of clinical suspicion. [27] Hypotension was diagnosed when mean blood pressure was lower than gestational age in weeks. [28] Necrotizing enterocolitis was classified according to the Modified *Bell’s* staging system. [29] Retinopathy of prematurity (ROP) was graded using the International Classification of ROP. [30] Periventricular leukomalacia was classified according to *De Vries et al.*. [31] Periventricular-intraventricular hemorrhage was graded using *Volpe*’s classification. [32] Hyaline membrane disease, pulmonary hemorrhage, neonatal seizures, mechanical ventilation and postnatal surfactant or corticosteroid administration were also assessed.

At 24 months of corrected age all children were evaluated for neurodevelopmental outcomes using the GMDS-III. [22] A global development quotient (GDQ) and development quotients for each specific area were calculated. Neuropsychomotor developmental delay was considered when GDQ was equal to or smaller than 70.

Cerebral palsy (CP) and vision and hearing impairment were also investigated. CP was diagnosed by a neuropaediatrician using the definition of the European Cerebral Palsy Network. [33] Vision and hearing impairment were systematically evaluated by a pediatric ophthalmologist and otolaryngologist.

Severe neurodevelopmental impairment was considered in the presence of at least one of the following: neuropsychomotor developmental delay, CP, and neurossensorial hearing impairment with need of implantable hearing device or blindness.

### Data analysis

Statistical analysis was performed using IBM®SPSS® Statistics version 26. Categorical variables are presented as frequencies and percentages, and continuous variables as means and standard deviations (SD) if normally distributed. Normal distribution was verified through the Kolmogorov-Smirnov test or skewness and kurtosis (maximum tolerated interval of -1 to 1). Bivariate analysis was performed using the χ2 test (or Fisher exact test as appropriate) for categorical variables and t test for continuous variables.

Logistic regression was performed to identify the predictors and outcomes of FGR. Quality of fit was verified by the Hosmer and Lemeshow test and significance by the Omnibus test. Linear regression was used to evaluate the impact of FGR on the neurodevelopmental outcome and to adjust for confounding variables with analysis of covariance. We constructed a model by adjusting for statistically significant and relevant maternal and perinatal factors. In this model we excluded variables with significant collinearity and with a very small number of cases (placenta previa, chorioamnionitis and cesarean delivery). Neonatal variables were not included in our model as they may be on the pathway between FGR and neurodevelopmental outcomes.

All reported p values are two-tailed with values inferior to 0.05 indicating statistical significance.

Approval was obtained from the local Ethics Committee (process number OBS.SF.40/2021).

## Results

**Fig. 1:**
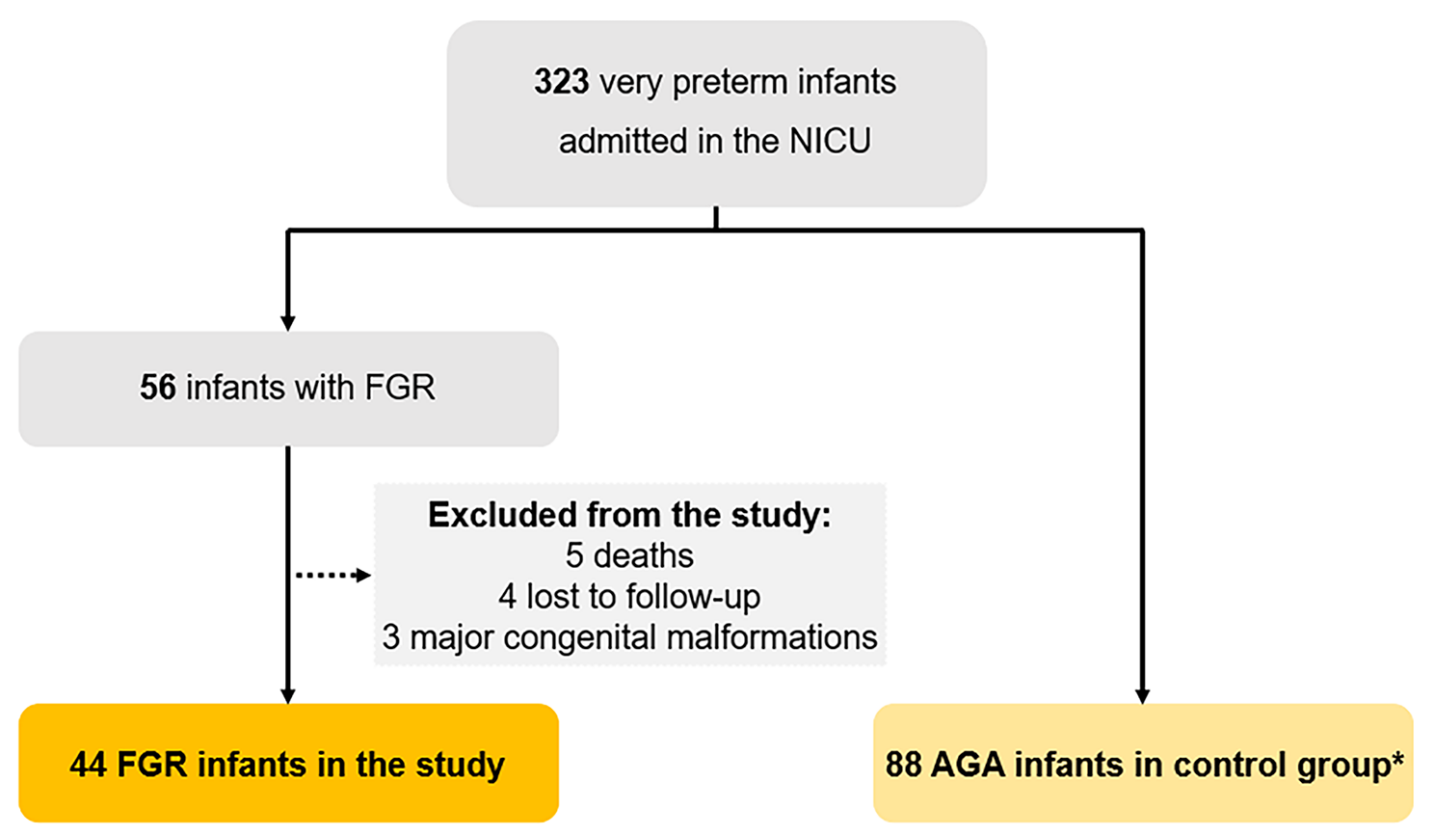
Flowchart of patient selection. AGA, appropriate for gestational age; FGR, fetal growth restriction; NICU, neonatal intensive care unit. *Selected with a proportion of 2 controls for 1 case and matched for gestational age and date of admission in the NICU. The remaining very preterm infants were not included in the study

During the study period 323 very preterm infants were admitted in the NICU. Of these, 56 (17.3%) were FGR infants. Twelve FGR infants were excluded, 5 (8.9%) due to death before discharge. Final sample size was 132 infants: 44 (33.3%) with FGR and 88 (77.7%) in the control group (Fig. 1).

### Maternal, perinatal and neonatal characteristics

Mean gestational age at birth was 29.09 ± 1.36 weeks in the FGR group and 29.15 ± 1.56 weeks in the control group (p = 0.837). Average birthweight was lower in the study group (856.48 ± 201.66 gram (g) vs. 1310.86 ± 280.14 g in the control group, p < 0.001). Remaining baseline characteristics are shown in Table 1.

**Table 1:** Characteristics of overall sample

	All infants (n = 132)	FGR infants (n = 44)	AGA infants (n = 88)	*p* value^*^
**Maternal characteristics**				
Maternal age – mean ± SD [years]	31.98 ± 5.69	32.11 ± 5.95	31.91 ± 5.60	0.847
Nulliparous – n (%)	89 (67.4)	29 (65.9)	60 (68.2)	0.793
Placenta previa – n (%)	5 (3.8)	1 (2.3)	4 (4.5)	0.664
Chorioamnionitis – n (%)	6 (4.5)	0 (0.0)	6 (6.8)	0.191
Hypertension/preeclampsia – n (%)	56 (42.4)	32 (72.7)	24 (27.3)	**< 0.001**
HELLP syndrome – n (%)	14 (10.6)	8 (18.2)	6 (6.8)	0.069
Gestational diabetes – n (%)	16 (12.1)	3 (6.8)	13 (14.8)	0.187
Thrombophilia – n (%)	13 (9.8)	6 (13.6)	7 (8.0)	0.359
Autoimmune disease – n (%)	9 (6.8)	2 (4.5)	7 (8.0)	0.717
Smokers – n (%)	32 (24.2)	13 (29.5)	19 (21.6)	0.433
Education level – n (%)				0.689
Lower secondary Upper secondary Tertiary	38 (28.8) 36 (27.3) 58 (43.9)	14 (31.8) 10 (22.7) 20 (45.5)	24 (27.3) 26 (29.5) 38 (43.2)	
**Perinatal characteristics**				
Pregnancy surveillance – n (%)				0.863
Full Late onset No surveillance	125 (94.7) 5 (3.8) 2 (1.5)	42 (95.5) 2 (4.5) 0 (0.0)	83 (94.3) 3 (3.4) 2 (2.3)	
Twin – n (%)	15 (11.4)	5 (11.4)	10 (11.4)	1.000
Antenatal corticosteroid – n (%)	109 (82.6)	38 (86.4)	71 (80.7)	0.417
Antenatal neuroprotection – n (%)	50 (37.9)	17 (38.6)	33 (37.5)	0.480
Labor induction – n (%)	84 (63.6)	44 (100)	40 (45.5)	**< 0.001**
PPRM – n (%)	20 (15.2)	0 (0.0)	20 (22.7)	**0.002**
Cesarean delivery – n (%)	98 (74.2)	44 (100)	54 (61.4)	**< 0.001**
Outborn – n (%)	9 (6.8)	0 (0.0)	9 (10.2)	0.069
Gestational age – mean ± SD [weeks]	29.13 ± 1.49	29.09 ± 1.36	29.15 ± 1.56	0.837
Birthweight – mean ± SD [gram]	1159.40 ± 334.21.	856.48 ± 201.66	1310.86 ± 280.14	**< 0.001**
Male – n (%)	69 (52.3)	21 (47.7)	48 (54.5)	0.460
5-minute Apgar score < 7 – n (%)	7 (5.3)	3 (6.8)	4 (4.5)	0.686
Endotracheal intubation – n (%)	28 (21.2)	13 (29.5)	15 (17.0)	0.098
**Neonatal characteristics**				
CRIB score > 5 at admission – n (%)	24 (18.2)	21 (47.7)	3 (3.4)	**< 0.001**
Late-onset sepsis – n (%)	29 (22.0)	16 (36.4)	13 (14.8)	**0.005**
Hyaline membrane disease – n (%)	45 (34.1)	19 (43.2)	26 (29.5)	0.119
Bronchopulmonary dysplasia – n (%)	6 (4.5)	5 (11.4)	1 (1.1)	**0.016**
Pulmonary hemorrhage – n (%)	2 (1.5)	2 (4.5)	0 (0.0)	0.188
Patent arterial duct – n (%)	32 (24.2)	12 (27.3)	20 (22.7)	0.566
Hypotension – n (%)	9 (6.8)	5 (11.4)	4 (4.5)	0.159
Necrotizing enterocolitis – n (%)	3 (2.3)	2 (4.5)	1 (1.1)	0.258
ROP grade ≥ 3 – n (%)	4 (3.0)	3 (6.8)	1 (1.1)	0.108
PVL grade ≥ II – n (%)	5 (3.8)	1 (2.3)	4 (4.5)	0.664
PIVH grade III or PVHI – n (%)	6 (4.5)	0 (0.0)	6 (6.8)	0.191
Seizures – n (%)	2 (1.5)	0 (0.0)	2 (2.3)	0.842
Mechanical ventilation – n (%)	49 (37.1)	19 (43.2)	30 (34.1)	0.308
Postnatal surfactant – n (%)	45 (34.1)	19 (43.2)	26 (29.5)	0.119
Postnatal corticosteroids – n (%)	6 (4.5)	6 (13.6)	0 (0.0)	**0.002**

^*^p value comparing FGR infants and AGA infants. AGA, appropriate for gestational age; CRIB, clinical risk index for babies; FGR, fetal growth restriction; HELLP, hemolysis, elevated liver enzymes, and low platelet count; PIVH, periventricular-intraventricular hemorrhage; PVHI, periventricular hemorrhagic infarction; PVL, periventricular leukomalacia; PPRM, prolonged premature rupture of membranes; ROP, retinopathy of prematurity; SD, standard deviation

**Fig. 2:**
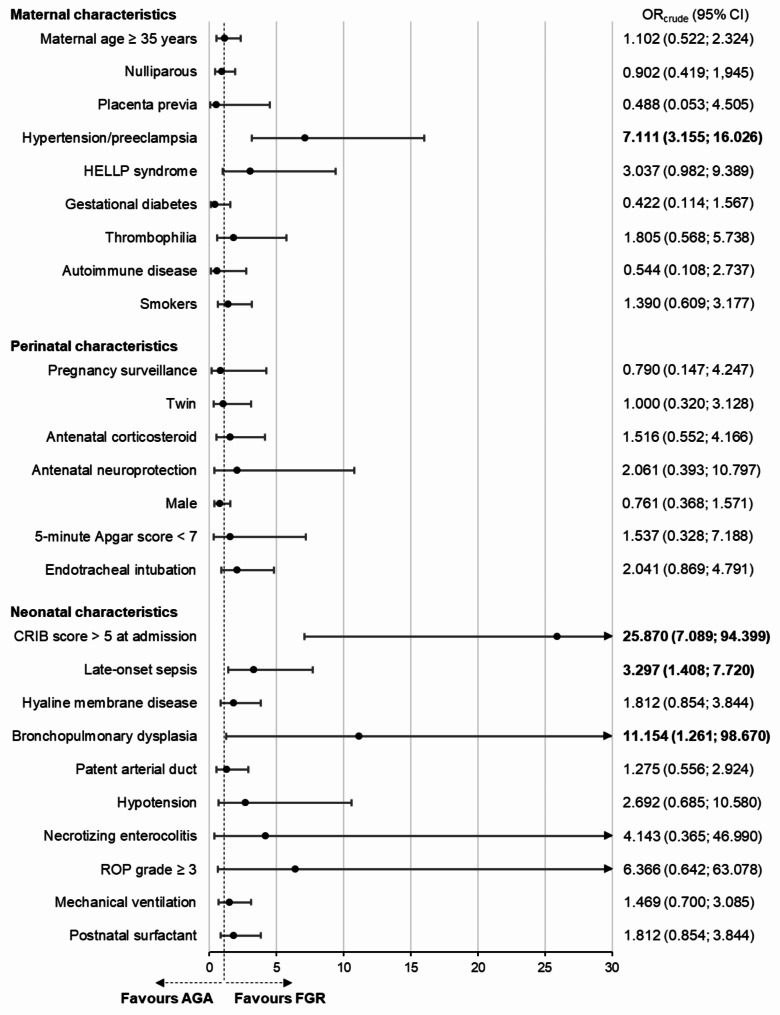
Maternal and perinatal predictors and neonatal outcomes of FGR. AGA, appropriate for gestational age; CRIB, clinical risk index for babies; FGR, fetal growth restriction; HELLP, hemolysis, elevated liver enzymes, and low platelet count; ROP, retinopathy of prematurity

Pregnant women carrying a FGR infant had a higher odd of having hypertension/preeclampsia during the pregnancy (OR 7.11, 95% confidence interval (CI), 3.16 to 16.03; p < 0.001). Additionally, these infants had a higher odd of having a CRIB score at NICU admission higher than 5 (OR 25.87, 95% CI, 7.09 to 94.40; p < 0.001), LOS (OR 3.30, 95% CI, 1.41 to 7.72; p = 0.006) and BPD (OR 11.15, 95% CI, 1.26 to 98.67; p = 0.030) (Fig. 2).

### Neurodevelopmental follow-up at 24 months of corrected age

Overall risk of severe neurodevelopmental impairment in the FGR infants was 11.4% and 3.4% in the control group (p = 0.116). No FGR infant was diagnosed with CP (vs.2.3% control, p = 0.842). Only one FGR child (2.3%) had neurosensorial hearing impairment with need of implantable hearing device (vs. none in control group, p = 0.671). No blindness was present in both groups.

Mean GDQ was lower for FGR infants, and after adjusting for confounding, FGR maintained its negative impact on the GDQ (adjusted coefficient: -16.703; p = 0.009). The overall scores for the five neurodevelopmental domains were highest for personal-social-emotional skills (107.02 ± 16.34), followed by eye and hand coordination skills (105.61 ± 14.20) and foundation of learning skills (102.23 ± 13.74), and were lowest for gross motor skills (97.90 ± 11.88) and language and communication skills (96.39 ± 18.88). FGR had a significant impact on all domains except for gross motor skills, even though the score was numerically lower than the average. After adjusting for both maternal and perinatal factors, FGR continues to have a significant adverse impact on language and communication skills, eye and hand coordination skills and foundation of learning skills (Table 2).

**Table 2:** Impact of fetal growth restriction in the global development quotient (GDQ) and in the different neurodevelopmental domains at 24 months of corrected age

	Mean SD	β^a^ (95% CI)	*p* value*	β_adjusted_^b^ (95% CI)	Standardized β^c^	*p* value*
**Global development quotient**						
FGR	96.39 ± 18.68	-7.625 (-12.758; -2.492)	**0.004**	-16.703 (-28.915; -4.492)	-0.525	**0.009**
AGA	104.01 ± 11.07	Ref		Ref		
All infants	101.47 ± 14.46					
**Gross motor skills**						
FGR	95.61 ± 14.32	-3.432 (-7.746; 0.882)	0.118	-6.781 (-16.054; 2.491)		0.146
AGA	99.05 ± 10.35	Ref		Ref		
All infants	97.90 ± 11.88					
**Personal–social–emotional skills**						
FGR	102.66 ± 21.52	-6.545 (-12.429; -0.662)	**0.030**	-11.065 (-22.782; 0.653)		0.063
AGA	109.20 ± 12.60	Ref		Ref		
All infants	107.02 ± 16.34					
**Language and communication skills**						
FGR	88.61 ± 22.78	-11.670 (-18.292; -5.049)	**0.001**	-21.924 (-38.822; -5.025)	-0.546	**0.013**
AGA	100.28 ± 15.32	Ref		Ref		
All infants	96.39 ± 18.88					
**Eye and hand coordination skills**						
FGR	102.07 ± 18.62	-5.318 (-10.442; -0.194)	**0.042**	-15.446 (-27.658; -3.234)	-0.480	**0.015**
AGA	107.39 ± 11.07	Ref		Ref		
All infants	105.61 ± 14.20					
**Foundations of learning skills**						
FGR	98.18 ± 17.70	-6.068 (-10.995; -1.141)	**0.016**	-15.211 (-26.916; -3.506)	-0.481	**0.013**
AGA	104.25 ± 10.82	Ref		Ref		
All infants	102.23 ± 13.74					

^a^ Coefficients from unadjusted model. ^b^ Coefficients from model adjusted for maternal and perinatal factors. ^c^ Standardized coefficients from adjusted model. ^*^p value comparing FGR infants and AGA infants. GDQ, global development quotient; Ref, reference; SD, standard deviation

## Discussion

### Main findings

Our findings suggest that (1) in severe preterm infants, FGR is associated with a statistically significant increased risk of poor neurodevelopmental outcome at 24 months old of corrected age and (2) its impact on the different neurodevelopmental domains varies, with a greater impact in language and communication, eye and hand coordination and foundation of learning skills.

The impact of FGR on neurodevelopment is in line with those of some previous studies. [15, 20, 34–36] Of note, a meta-analysis by Sacchi et al. showed poorer cognitive function during the first 12 years of life in children who had FGR and were SGA compared with AGA children matched for gestational age. [13] Nevertheless, the criteria of FGR have been extensively debated and as such, the criteria used in previous studies differ. We believe that by using the definition established in the most recent consensus using a Delphi procedure we were able to exclusively study FGR infants and exclude SGA infants that were previously wrongly included in the first group. [19, 37–41].

Regarding the impact on the different domains, few studies have addressed the influence of FGR. One study showed that children born before 27 weeks of gestation with birthweight lower than the 10th centile had worse fine motor and social interaction skills at 2 years of corrected age than age-matched AGA infants. [11] Another study demonstrated that children born at 27–34 weeks of gestation with FGR were particularly impaired in cognitive, behavioural and hearing development domains during the first 12 years of life compared with age-matched non-FGR infants. Nevertheless, the aforementioned papers significantly differ from our study regarding the FGR definition, the selected control group, the assessment ages, and the follow-up assessment tools. Hence, the comparison between our results and the findings of these other papers must be interpreted with caution.

The mechanisms behind FGR are not fully understood. Maternal vascular malperfusion as a consequence of anomalous remodelling of the uterine spiral arteries is believed to be the most frequent factor leading to placental insufficiency, with subsequent hypoxia-reoxygenation damage. [42] Inflammatory lesions and villitis of unknown origin may also play a role. [42, 43].

The pathophysiology of FGR appears to impact only specific neurodevelopmental domains. Placental insufficiency inherent to FGR has been associated with metabolic and structural changes in the fetal and postnatal brain [19, 37–41] mainly due to oxidative stress and an adaptive response to malperfusion with shunt to specific organs such as the brain. [44, 45] This abnormal brain flow persists in the first days of the postnatal period, potentiating hyperoxia and perpetuating oxidative stress. [44, 46] Some authors have observed smaller head circumferences in children born with FGR, [9, 10, 19, 40] a finding that has been correlated with worse cognitive and language outcomes. [19, 20] Decreased brain volumes in FGR infants *in utero*, [37] at preschool [38, 39] and early school ages [40] have also been reported as well as an altered distribution pattern of grey and white matter. [34, 37, 38] We postulate that our findings may be justified by these asymmetries in brain development that may preferentially affect specific brain structures.

Finally, we found no association between FGR and the risk of having CP, neurosensorial hearing impairment with need of implantable hearing device or blindness. Other studies were also unable to show this association. [11, 12, 15, 34] Our results could be explained by the small sample size and the low prevalence of these outcomes in our sample.

### Strengths and limitations

To the best of our knowledge, this is the first study that aims to determine the impact of FGR on neurodevelopmental outcomes using the criteria defined by the international Delphi consensus. [5] Thus, by using fetal growth references instead of postnatal references (as most available studies have done) we believe we estimated the real impact of FGR on child health and neurodevelopmental outcomes.

Our study also included neurodevelopment assessments using GMDS-III performed by the same two trained educators throughout the study period who were blind to the FGR diagnosis, ensuring uniform evaluations. These scales have been extensively validated to assess the psychomotor development of preterm infants at an early age and are regarded as one of the most accurate infant developmental tests in Europe and Portugal, particularly in the follow-up of at-risk infants. [23, 47–49].

This study also has some limitations. The study was conducted in a single tertiary maternity, and as such, these findings may be skewed and must be interpreted with caution. Nevertheless, given the high rates of stillbirths and the elevated neonatal mortality among very preterm FGR infants, this limitation is practically unsurmountable in all studies. [7, 8, 34, 50] As a retrospective study, diagnoses and comorbidities may have been underreported. However, since our institution participates in the prospective National Registry of Very Preterm Newborns and the eNewborn European Network database, [51] we believe the data included in our study was precise and accurate. In addition, the non-random selection of controls and FGR infants may have led to bias. Nevertheless, we believe the methodology used for selecting the controls and studying population differences by adjusting for confounding variables minimized this issue to the extent possible. While we believe the study was adequately powered for the primary endpoint, the small sample size and low frequency of some of the outcomes (such as cerebral palsy and blindness) may facilitate type II error when analysing comorbidities. Finally, although we adjusted our results for confounding variables, other unmeasured confounders may have influenced the final results.

In conclusion, FGR constitutes a significant risk for neurodevelopmental impairment during childhood. Both maternal and perinatal factors play an essential role in its development. Efforts should be made to ensure early and correct diagnosis of FGR and all contributing factors, with the aim of reducing their adverse impact on neurodevelopmental outcomes. Further prospective and multicentric follow-up studies with standardized definitions are crucial to expand our understanding of the impact of FGR on the neurodevelopment outcome of very preterm infants and the underlying mechanisms.

## Data Availability

All data generated or analysed during this study are included in this published article.

## List of Abbreviations and Acronyms

AGA: Appropriate for gestational age
BPD: Bronchopulmonary dysplasia
CP: Cerebral palsy
CRIB: Clinical risk index for babies
FGR: Fetal growth restriction
GDQ: Global development quotient
GMDS-III: Griffiths III Mental Development Scale
HELLP: Hemolysis, elevated liver enzymes, and low platelets
HMD: Hyaline membrane disease
LOS: Late-onset sepsis
NEC: Necrotizing enterocolitis
NICU: Neonatal Intensive Care Units
PIVH: Periventricular-intraventricular hemorrhage
PPRM: Prolonged premature rupture of membranes
PVHI: Periventricular hemorrhagic infarction
PVL: Periventricular leukomalacia
ROP: Retinopathy of prematurity
SD: Standard deviation
SGA: Small for gestational age
TORCH: Toxoplasmosis, rubella, cytomegalovirus, and herpes simplex virus
